# Dissection of the Ovulatory Process Using *ex vivo* Approaches

**DOI:** 10.3389/fcell.2020.605379

**Published:** 2020-12-09

**Authors:** Alexander A. Tokmakov, Vasily E. Stefanov, Ken-Ichi Sato

**Affiliations:** ^1^Faculty of Life Sciences, Kyoto Sangyo University, Kyoto, Japan; ^2^Department of Biochemistry, Saint Petersburg State University, Saint Petersburg, Russia

**Keywords:** oocyte, ovulation, maturation, follicle rupture, *in vitro*

## Abstract

Ovulation is a unique physiological phenomenon that is essential for sexual reproduction. It refers to the entire process of ovarian follicle responses to hormonal stimulation resulting in the release of mature fertilization-competent oocytes from the follicles and ovaries. Remarkably, ovulation in different species can be reproduced out-of-body with high fidelity. Moreover, most of the molecular mechanisms and signaling pathways engaged in this process have been delineated using *in vitro* ovulation models. Here, we provide an overview of the major molecular and cytological events of ovulation observed in frogs, primarily in the African clawed frog *Xenopus laevis*, using mainly *ex vivo* approaches, with the focus on meiotic oocyte maturation and follicle rupture. For the purpose of comparison and generalization, we also refer extensively to ovulation in other biological species, most notoriously, in mammals.

## Overview of Ovulation

Oocytes of most vertebrate species, including frog oocytes, reside and grow in the ovaries while arrested in the first meiotic prophase. At the advanced stages of growth, oocytes resting in ovarian follicles are surrounded by the follicle envelope, consisting of several layers, such as theca/epithelial layer, granulosa cell layer, and vitelline envelope. Direct contacts between the oocyte and somatic cells in the follicle are formed via terminal gap junctions connecting the oocyte and follicle cell plasma membranes (Browne et al., 1979). Communication and paracrine interactions between the oocyte and somatic cells in the follicle were found to be necessary for normal ovulation. The fully grown frog oocytes are not competent for fertilization. They are characterized by a low activity of the main meiotic regulators, the cytostatic factor (CSF) and maturation-promoting factor (MPF) (Masui and Markert, 1971; Smith and Ecker, 1971). MPF was originally defined by Masui and Markert as a cytoplasmic activity from mature oocytes that causes complete maturation upon injection into immature oocytes, and CSF, as a cytoplasmic activity from unfertilized eggs that promotes metaphase arrest upon transfer to early dividing embryos. It was found subsequently that MPF represents a complex of cyclin B and Cdk1 kinase (Hunt, 1989), and CSF was identified as a multicomponent system comprising the meiotic protein kinase Mos and the MAPK pathway (reviewed in Tunquist and Maller, 2003; Schmidt et al., 2006; Wu and Kornbluth, 2008).

**FIGURE 1:**
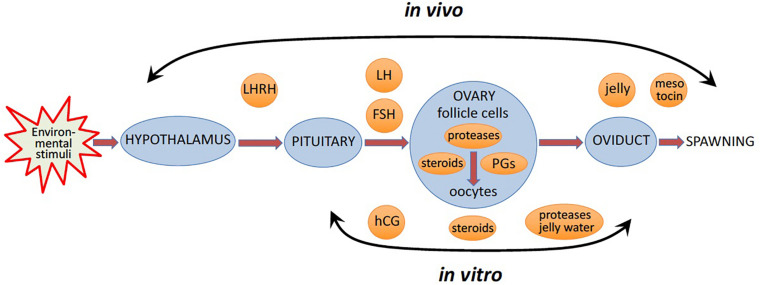
*In vivo* and *in vitro* ovulation of frog oocytes. *In vivo*, favorable environmental factors initiate a hormonal cascade via the hypothalamic-pituitary-gonadal axis, to cause oocyte ovulation resulting in egg spawning. *In vitro*, oocyte ovulation can be induced in the ovarian fragments or isolated ovarian follicles by pituitary extracts, or luteinizing hormone (LH), or human Chorionic Gonadotropin (hCG), a functional analog of LH, or multiple steroid hormones.

The fully grown frog oocytes acquire fertilization competence in the process of meiotic maturation. They progress through the meiotic cell cycle and arrest again before fertilization in the second meiotic metaphase with high activity of CSF and MSF. Hormonal stimuli release the prophase arrest and initiate oocyte maturation when oocytes rest in ovarian follicles. The maturing frog oocytes leave their follicles, advance from the ovary into the open coelomic body cavity, pass through the oviduct, and accumulate, for a short time, in the uterus. Finally, they are deposited outside the body into aquatic environments where fertilization occurs (Figure 1). Thus, the process of meiotic maturation is accompanied or immediately followed by oocyte release from the ovary, and the entire process of follicle responses to hormonal stimulation, which produces mature follicle-free fertilization-competent oocytes, is called “ovulation” (of note, in some studies the term “ovulation” is used narrowly, only for oocyte liberation from the ovarian follicles). Therefore, ovulation engages, as the major events, oocyte maturation and oocyte release from ovarian follicles. In addition, egg oviposition may also be considered as an integral event of ovulation in frogs because most amphibians are oviparous species with external fertilization.

**FIGURE 2:**
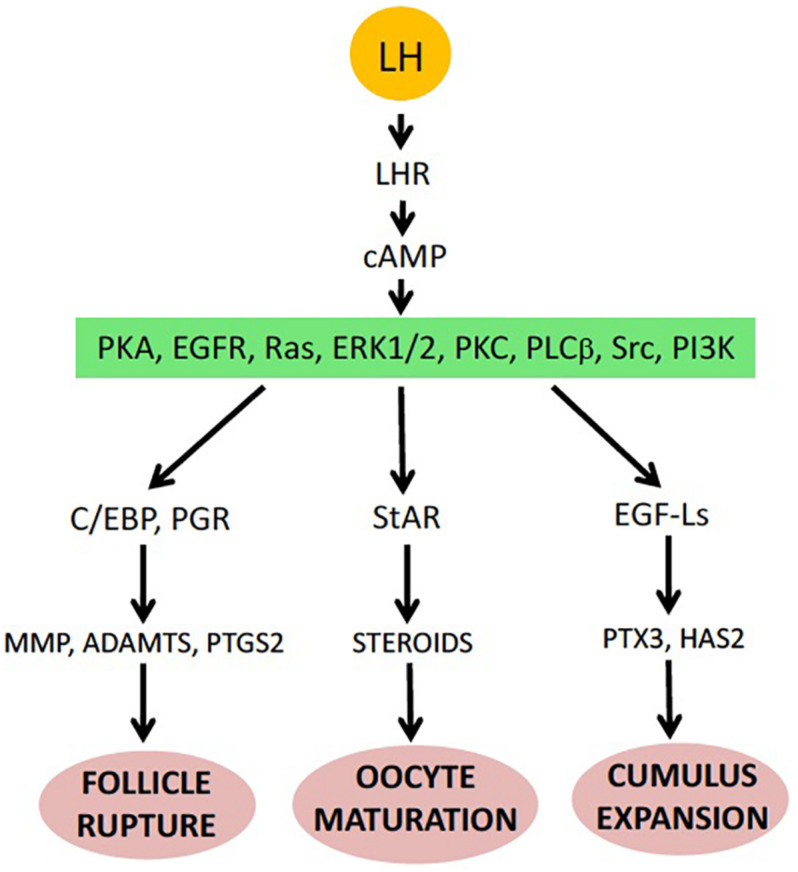
Major events of ovulation in the mammalian ovarian follicle (see details in the text).

In nature, the environmental factors stimulate production of luteinizing hormone releasing hormone (LHRH), also known as gonadotropin-releasing hormone (GnRH), by the hypothalamus (Figure 1). In response to LHRH, the pituitary produces gonadotropins, such as luteinizing hormone (LH) and follicle stimulating hormone (FSH). The hormones upregulate various genes involved in ovulation and stimulate steroidogenic ovarian follicle cells (granulosa and theca cells), surrounding an oocyte within a follicle, to produce various steroid hormones, prostaglandins (PGs), and multiple proteolytic enzymes (Figure 1). LH activates Gαs protein-coupled LH receptors (LHCGRs) expressed in follicular cells, leading to increased cAMP production and activation of PKA (Simoni et al., 1997; Wood and Strauss, 2002). In addition, LHCGR also activates Gq/11 and stimulates phospholipase C (PLC) and its downstream signaling in granulosa cells (Breen et al., 2013). Thus, LHCGR activation triggers multiple intracellular signal transduction pathways mediated by different signaling molecules. Involvement of PKA, PKC, PI3K, PLC, Src, EGFR, MAPK and Ras in response to LH and gonadotropin has been documented in mammalian follicles (Figure 2) (Jamnongjit and Hammes, 2006; Richards and Ascoli, 2018). In mammals, several transcription factors, such as PGR, PRARG, HIFs, CEBPA, RUNX1, RUNX2, NRIP1, NR5A2, become activated in follicular cells, resulting in the increased production of the enzymes that play important roles in the ovulatory process (Duffy et al., 2019). These enzymes cover the three crucial processes indispensable for successful ovulation, such as steroidogenesis, prostaglandin synthesis, and proteolysis.

Follicular steroid production and steroid-mediated signaling are critical for the initiation of oocyte maturation during the ovulatory process. Early studies in frogs demonstrated that the treatment of isolated amphibian ovarian follicles with frog pituitary homogenate (FPH) increases follicular progesterone (P4) levels, which, in turn, initiate oocyte maturation (Petrino and Schuetz, 1987). It was found using *in vitro* cultures of *Rana pipiens* and *Rana dybowskii* ovarian follicles, that steroid production in ovarian follicle cells is mediated by activation of the cAMP/PKA steroidogenic pathway. A marked increase in intrafollicular P4 levels to those produced by FPH occurred when frog ovarian follicles were exposed to exogenous dibutyryl cAMP and a phosphodiesterase inhibitor IBMX. The observed elevation of follicular levels of P4 caused by FPH administration or cAMP stimulation required the presence of somatic follicular cells (Kwon and Schuetz, 1986). It was concluded, that FPH increases the intracellular level of cAMP by modulating the relative activity of adenylyl cyclase and phosphodiesterase (Kwon and Schuetz, 1986; Kwon et al., 1990). The production of the three main steroids, such as P4, testosterone and estradiol, was reported to occur *in vitro* in the isolated *Xenopus* follicles stimulated by gonadotropin hormones (El-Zein et al., 1988; Lutz et al., 2001). Markedly, multiple steroid hormones were found to induce maturation of frog oocytes (Baulieu et al., 1978; Masui and Clarke, 1979). Their contribution into the steroid signaling during maturation is discussed further in the section “Meiotic resumption.”

Similarly to frogs, it was demonstrated that in mammals too the cAMP/PKA signaling cascade is the major pathway regulating steroid biosynthesis. In addition, it was found that, the steroidogenic acute regulatory protein (StAR), highly expressed in both theca and granulosa cells of mammalian follicles, is involved in steroidogenesis. Increased expression of StAR and upregulation of StaR activity via phosphorylation were shown to promote steroidogenesis, whereas inhibition of StAR expression led to a dramatic fall in steroid biosynthesis (Wood and Strauss, 2002; Jamnongjit and Hammes, 2006). This labile mitochondrial phosphoprotein mediates the transfer of cholesterol, the precursor for all steroid hormones, from the outer to inner mitochondrial membrane. This reaction represents the rate-limiting step in steroid hormone biosynthesis. A tight correlation between expression of the StAR protein and steroid synthesis has been reported (Manna et al., 2009). The cAMP/PKA pathway was established as a major signaling pathway for hormone-stimulated StAR expression, and the MAPK signaling cascade was demonstrated to mediate this process (Manna and Stocco, 2011). MAPK activation was found to elevate StAR expression in some steroidogenic cell lines (Gyles et al., 2001). Membrane-permeable cAMP analogs induce MAPK activation in granulosa cells of mammalian follicles, suggesting that gonadotropin-dependent activation of MAPK occurs downstream of cAMP/PKA signaling (Cameron et al., 1996; Das et al., 1996; Su et al., 2002). Notably, in contrast to frogs, synthesis of steroids is sufficient but not ultimately necessary for maturation of mammalian oocytes, indicating that higher vertebrates have developed redundant systems to trigger this process (Lieberman et al., 1976; Yamashita et al., 2003; Jamnongjit and Hammes, 2006).

Simultaneously with the elevation of follicular steroid production, which initiate meiotic oocyte maturation in frogs, LH upregulates a plethora of genes related to follicular rupture and oocyte liberation from the ovarian follicle. It was demonstrated using isolated ovarian fragments of *Xenopus laevis* that oocyte release from the follicles is a transcription-dependent process; inhibition of transcription with actinomycin D blocked hormone-induced follicle rupture, so that mature oocytes remained entrapped in the follicles (Liu et al., 2005; Sena and Liu, 2008). The major classes of genes related to follicle rupture and induced during ovulation via different transcription factors include the enzymes involved in PG synthesis and multiple ovarian proteases involved in decomposition of the follicle wall. The fact that inhibitors of eicosanoid synthesis can block ovarian collagenolysis and suppress gonadotropin-induced elevation in the content of interstitial collagenase in preovulatory mammalian follicles (Reich et al., 1991) strongly suggests involvement of prostaglandins upstream of follicular rupture. Indeed, prostaglandins were identified as the universal intrafollicular signaling molecules critical for ovulation in different biological species, including frogs and mammals (Schuetz, 1986; Chang et al., 1995; Sena and Liu, 2008; Niringiyumukiza et al., 2018; Takahashi et al., 2018).

Oocyte liberation from the ovary requires physical rupture of the ovarian follicle. This event is highly synchronized with meiotic resumption in oocytes. It was initially found that pre-ovulatory follicles contain a dense meshwork of collagen fibers, suggesting that collagenolysis might be a prerequisite for follicular rupture. Indeed, early morphological studies have revealed a decrease in collagen fiber contents in the apical wall of mammalian follicles prior to ovulation (Espey, 1967; Tsafriri et al., 1990). It was demonstrated that intrabursal injections of broad specificity protease inhibitors inhibited ovulation and degradation of ovarian collagen in a dose-dependent fashion, and that intrafollicular injection of metalloproteinase inhibitors completely blocked ovulation (Reich et al., 1985; Peluffo et al., 2011). Subsequently, a number of *in vivo* and *in vitro* studies have demonstrated that various follicular metalloproteinases and serine proteases are upregulated during ovulation (Figure 2). Synergetic action of multiple proteases with different substrate specificities was found to be necessary for effective degradation of the follicular wall. Involvement of proteolytic enzymes in follicle rupture has been well established in mammalian, fly and fish species, however little is known about the molecular mechanisms of follicle rupture in frogs.

Following follicle rupture, liberated frog oocytes migrate from the ovary into the oviduct, where they complete maturation and acquire a jelly layer that is necessary for successful fertilization (Figure 1). In addition, the vitelline membrane surrounding the oocytes is crippled by proteolytic enzymes within the first part of the oviduct, making this membrane penetrable to sperm at the time of fertilization (Heasman et al., 1991). It was demonstrated that frog eggs have to pass through the oviduct in order to become fertilizable (Diakow et al., 1988). Three distinct jelly coats are found in *Xenopus laevis* and as many as six in *Rana pipiens* (Freeman, 1968; Bonnell and Chandler, 1996). The jelly coats are synthesized in the oviduct and deposited sequentially on the egg during its travel through the oviduct (Bakos et al., 1990). Constituents of the egg jelly layers are essential for egg fertilization by incoming sperm. Eggs which are stripped of their jelly layers could not be fertilized, but the addition of solubilized jelly was found to partially restore fertilization capacity (Olson and Chandler, 1999). Finally, an oxytocin family peptide hormone (mesotocin in frogs), which is released by the posterior pituitary, contributes at the stage of oviposition (Schmidt, 1984; Browne and Figiel, 2019; Figure 1). In frogs, the hormone is involved in the physical process of spawning and mating behavior. Oxytocin family hormones have uterus-contracting (uterotonic) activity that causes muscle contractions via a G protein- and calcium-mediated pathway and promotes oviposition (Jurek and Neumann, 2018). It was found in monkey and cow that follicular granulosa cells produce oxytocin in response to pre-ovulatory upsurge in LH (Voss and Fortune, 1991; Einspanier et al., 1997).

## *In vitro* Ovulation Models

In the wild, favorable environmental factors, such as balmy temperature, ample rainfall, protracted daytime, presence of males, etc., initiate oocyte ovulation in frogs via a hormonal cascade involving the hypothalamus, the pituitary, and ovarian follicle cells, as described in detail in the previous section (Figure 1). Normally, during the reproductive season, egg deposition and fertilization occur within several hours of hormonal stimulation. However, some eggs can be retained in the frog genital tract for much longer time and degrade there by an apoptotic process (Iguchi et al., 2013; Tokmakov et al., 2018). It was observed that aging frogs retained a larger number of ovulated eggs for a longer period than the young animals (Iguchi et al., 2013). It was also reported that decreasing temperatures could cause retention of mature eggs in the uterus for several days (Witschi, 1952). Thus, seasonal, environmental, and individual-specific factors make it difficult to control ovulation of frog oocytes *in vivo*. Of note, the same factors were also demonstrated to control the ovulatory process in different fish species, most notably, teleost fishes that have external fertilization similarly to frogs (Munakata and Kobayashi, 2010).

To facilitate dissection of the ovulatory process, various *in vitro* growth and ovulation models have been developed in different animals. Most of them involve the mammalian species, such as rodents, cows, pigs, horses, primates and even humans, where the studies would have apparent applications for assisted reproduction (Picton et al., 2003; Gerard and Robin, 2019; Telfer, 2019; Telfer et al., 2019). The development of technologies to grow and mature oocytes is very attractive for research, animal production technology and clinical practice. The ultimate goal of this approach is to grow, mature, ovulate and fertilize oocytes *in vitro* with the following production of viable embryos. The *in vitro* reproductive technologies were very successful in mammals. For instance, it was reported that the whole ovarian cell cycle can be reconstructed *in vitro* using the follicle culture of murine and human follicles (Skory et al., 2015). *In vitro* systems that support full development of murine oocytes starting from primordial germ cells or from induced pluripotent cells have been recently described (Hikabe et al., 2016; Morohaku et al., 2016). However, in frogs, it has proved difficult to grow early stage follicles to maturity *in vitro*. In fact, no report has been provided so far about the successful *in vitro* growth of frog oocytes. However, *in vitro* maturation and/or ovulation of frog oocytes have been consistently observed in the ovarian fragments and isolated fully grown follicles treated with homologous pituitary extracts (Heilbrunn et al., 1939; Wright, 1945; Wright, 1961; Dettlaff et al., 1964). Also, LH and human Chorionic Gonadotropin (hCG), the hormones that activate the same heptahelical Luteinizing Hormone/Choriogonadotropin Receptor (LHCGR), as well as various steroid hormones were found to be effective in inducing *in vitro* maturation of frog oocytes in ovarian fragments (Wright, 1961; Subtelny et al., 1968; Figure 1). Furthermore, it was reported that a number of streroids could induce GVBD in isolated *Rana pipiens* follicles even though follicle rupture did not occur (Schuetz, 1967). In addition, it was found that physiological meiotic maturation can be induced *in vitro* with some steroid hormones in *Rana pipiens* oocytes removed from their ovarian follicles. It was noted that the hormones work equally well with ovarian fragments or with isolated follicles (Smith et al., 1968). The early studies of *in vitro* ovulation and maturation in frogs were summarized and extensively reviewed by Smith and Ecker (1970).

Of note, frogs of the genus *Rana*, such as *Rana pipiens*, *Rana dybowskii* and *Rana japonica*, had been used most often during the early investigations of frog oocyte maturation. However, more recently, highly aquatic frogs of the *Xenopus* genus, such as *Xenopus laevis* and *Xenopus tropicalis*, have been employed as the main biological models in oocyte maturation and ovulation studies, focusing on signaling pathways and molecular machinery of these processes. In fact, the *Xenopus* pregnancy test, also called the Hogben test, based on the experiments suggested by Hogben and successively carried out by his students and co-workers in the 1930s (Shapiro and Zwarenstein, 1934), was used around the world for about three decades, until immunological test kits replaced it in the 1960s. In the test, female frogs were injected with the woman’s urine, and if the frogs had ovulated due to a high level of hCG in the urine, that meant the woman who provided the urine was pregnant.

Importantly, *in vitro* matured and ovulated frog eggs still cannot be fertilized efficiently because they are not penetrable to sperm. These eggs have the intact vitelline membrane, which is naturally degraded during physiological ovulation by proteolytic enzymes within the first part of the oviduct. In addition, *in vitro* ovulated frog eggs miss the jelly layer synthesized in the oviduct and deposited sequentially on the egg during its travel through the oviduct (Figure 1). Two general approaches have been developed that allow successful fertilization of *in vitro* matured frog eggs. One of them involves transferring investigated eggs into the body cavity of a host female frog, to let them pass through the frog’s oviducts (Lavin, 1964; Brun, 1975). Evidently, this approach represents a hybrid *in vitro/in vivo* technique, and it was extensively used in the studies of maternally inherited molecules by specifically removing or overexpressing particular mRNAs and proteins. For instance, the host-transfer technique was successfully used by the group of Janet Heasman to establish the essential role of Wnt signaling in *Xenopus* axis formation (Zuck et al., 1998; Mir and Heasman, 2008). Another approach to obtain *in vitro* matured fertilizable frog eggs involves enzymatic and/or manual removal of the vitelline membrane. Complete removal of the vitelline membrane can be carried out by combined treatment of eggs with pepsin and cysteine followed by manual removal of the loosened membrane (Kloc et al., 1989; Heasman et al., 1991). Extracts of diffusible components of *Xenopus* eggs jelly layers prepared by incubation of freshly ovulated eggs in high-salt buffers were shown to promote sperm’s ability to fertilize dejellied eggs in a dose-dependent manner (Olson and Chandler, 1999). It was reported that removal of the vitelline membrane and addition of the conditioned medium containing solubilized jelly obtained by soaking jellied eggs in buffer and Ficoll can vastly boost success rates of fertilization (Heasman et al., 1991; Olson and Chandler, 1999). For example, injection of oligonucleotides into isolated oocytes, followed by their *in vitro* maturation and fertilization using the abovementioned approach allowed to investigate the role played by xlgv7 mRNA in early development of the *Xenopus* embryo (Kloc et al., 1989).

## Maturation

Frog oocytes have been extensively used to study maturation and meiotic progression. In fact, most of the control mechanisms that operate in meiosis, including MPF and CSF, were first established in frogs. A number of key signaling molecules, such as membrane receptors, protein kinases, protein phosphatases, their substrates, inhibitors and activators, adaptor proteins, etc., have been characterized. Many of these studies were carried out *in vitro*, using isolated fully grown *Xenopus* oocytes treated with gonadotropins or steroid hormones (Figure 1). Especially, denuded frog oocytes, i.e., oocytes devoid of the follicle layer, proved to be very helpful in the maturation studies. Defolliculation could be accomplished by either enzymatic treatment or by manual dissection of oocytes from their ovarian follicles (Masui, 1967; Smith and Ecker, 1969). Also, the techniques of oocyte denucleation, as well as cytoplasmic and nuclear transfer (Dettlaff et al., 1964; Smith and Ecker, 1970; Masui and Markert, 1971), contributed greatly to the studies of molecular mechanisms of hormonal action, identification of MPF and CSF, and establishing their role in meiotic maturation.

### Diplotene Arrest in Immature Oocytes

Before maturation, fully grown *Xenopus* oocytes of the stage VI are naturally arrested at the diplotene stage of the first meiotic prophase at the G2/M boundary. They have an intact nuclear envelope, partially decondensed chromatin, high activity of transcription, high level of intracellular cAMP, and low activity of MPF and CSF. Several studies have implicated a constitutively activated G protein-coupled receptor 3, GPR3, as one of the key molecules responsible for elevated intracellular cAMP and maintaining meiotic prophase arrest in frog and mammalian oocytes (Mehlmann et al., 2004; Hinckley et al., 2005; Deng et al., 2008; Rios-Cardona et al., 2008). Both Gαs and Gβγ - mediated signaling were found to be involved in maintaining elevated cAMP levels and diplotene arrest in immature oocytes (Gallo et al., 1995; Lutz et al., 2000; Freudzon et al., 2005; Sheng et al., 2005). More recent studies in mammals demonstrated that cGMP too is a key regulator of meiotic transition in mammalian follicular oocytes (Jaffe and Egbert, 2017). It was found that mural granulosa cells of mammalian follicles contain a high content of cGMP in a quiescent stage due to the high expression levels of the transmembrane guanylyl cyclase natriuretic peptide receptor 2 (NPR2) and its ligand the natriuretic peptide C (NPPC) (Vaccari et al., 2009; Zhang et al., 2010). cGMP was found to diffuse from the somatic compartment into oocytes through gap junctions and inhibit phosphodiesterase 3A (PDE3A) to maintain a high level of cAMP and meiotic arrest in the oocytes (Norris et al., 2009; Vaccari et al., 2009). It should be noted that involvement of NPPC-NRP2 autocrine regulatory mechanism in frog ovulation has not been demonstrated yet.

**FIGURE 3:**
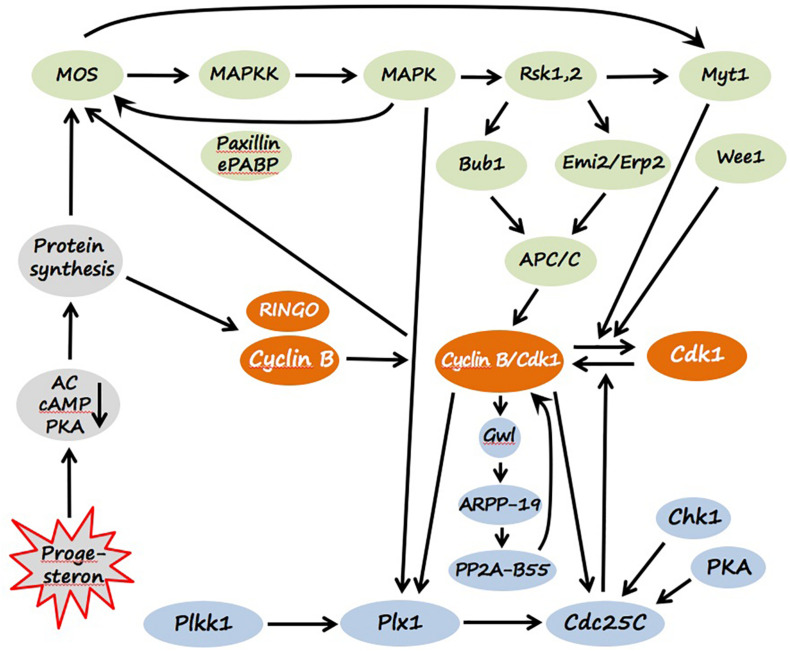
Signaling pathways of *Xenopus* oocyte maturation. Molecular components of CSF and MPF are colored green and orange, correspondingly. The components of MPF auto-amplification loops mediated by polo-like kinase (Plx1) and Greatwall kinase (Gwl) are presented in light blue, and the factors involved in the initiation of egg maturation are shown in gray. Detailed explanations are provided in the text (see section “Maturation”).

In prophase-arrested oocytes, the major bulk of Cdk1, a key component of MPF, is present in a free monomeric and low-activity form, and some part of the kinase is stored as an inactive Cdk1/Cyclin B complex, called pre-MPF. The inhibitory kinase Myt1, which phosphorylate Cdk1 on Thr14 and Tyr15, plays a major role in maintaining pre-MPF in the inactive state (Nakajo et al., 2000). In addition, Chk1 kinase, contributes to maintaining low activity of MPF in immature oocytes. The kinase inhibits the MPF-activating phosphatase Cdc25C through direct phosphorylation on Ser28 (Nakajo et al., 1999; Figure 3). Markedly, a direct link between PKA activated by a high level of intraoocyte cAMP and Cdc25 has been reported, indicating that PKA phosphorylates and inactivates Cdc25 in frog oocytes (Duckworth et al., 2002; Shibuya, 2003). Besides the low activity of MPF, the lack of active CSF is also crucial for maintaining prophase arrest. Low activity of CSF, and specifically of the MAPK cascade, in immature prophase-arrested oocytes is ensured by the absence of Mos protein (Figure 3) that is synthesized from maternal mRNA later during maturation. Notably, CSF and MPF are embedded in a loop of positive feedback, so they can mutually affect each other’s activity (Ferrell, 2002). Low activity of the two key meiotic regulators and the existence of positive feedback between them secure stability of the prophase I arrest in immature oocytes.

### Meiotic Resumption

Stimulation of isolated *Xenopus* follicles by gonadotropins was reported to increase production and release of several steroids, such as P4, testosterone, and estradiol, by steroidogenic follicle cells (Fortune and Tsang, 1981; Fortune, 1983; El-Zein et al., 1988). Importantly, either P4 or testosterone, but not estradiol and other estrogens, were shown to be able to induce *in vitro* maturation of frog oocytes. P4 was suggested to play a major role in triggering maturation of frog oocytes because its intra-oocyte concentration increases robustly to micromolar levels during meiosis re-entry (Haccard et al., 2012), and because it can induce meiotic maturation of isolated denuded frog oocytes at submicromolar and low micromolar concentrations (Jacobelli et al., 1974; Reynhout et al., 1975). It was noted that induction of maturation by exogenous P4 is significantly faster and more efficient in defolliculated oocytes than in the follicles, suggesting that follicle layer may not be well permeable for the hormone (Haccard et al., 2012; Tokmakov et al., 2019). In addition, evidence has been presented that androgens, rather than progestogens, might be the *bona fide* effectors that promote maturation of *Xenopus* oocytes via classical androgen receptor (Lutz et al., 2001; Hammes, 2004; Evaul et al., 2007; Sen et al., 2011). Testosterone was identified as the main steroid produced quantitatively in response to LH and capable of inducing *in vitro* maturation as efficiently as P4 (Baulieu et al., 1978; Fortune and Tsang, 1981; El-Zein et al., 1988; Lutz et al., 2001). It was also suggested that both types of steroids can be involved in meiotic maturation of *Xenopus* oocytes, considering high intra-oocyte levels of CYP17, an enzyme converting progestogens to androgens (Yang et al., 2003; Deng et al., 2009).

One of the earliest responses of oocytes to P4 is a decrease in the intracellular level of cAMP due to inhibition of plasma membrane adenylate cyclase (AC) (Schorderet-Slatkine et al., 1978; Finidori-Lepicard et al., 1981). The reduction of cAMP concentration and subsequent decline in PKA activity are crucial for initiating maturation. Microinjections of PKA catalytic subunit block P4-induced maturation, and injecting PKA inhibitors induce oocyte maturation in the absence of P4 (Maller and Krebs, 1977; Huchon et al., 1981). The finding that P4 can initiate oocyte maturation only when applied externally but not when microinjected into the cytoplasm or the nucleus, and the fact that MPF activation is observed in enucleated oocytes (Masui and Markert, 1971; Smith and Ecker, 1971) indicated that P4 receptor (PGR) is not a conventional nuclear steroid receptor but rather a non-transcriptional membrane-associated signaling receptor. Multiple studies suggest that inhibition of AC is mediated by the G-protein coupled transmembrane receptor GPR3 via a “release of inhibition” mechanism. It was shown that overexpression of GPR3 suppressed steroid-induced maturation of isolated *Xenopus* oocytes and gonadotropin-induced maturation of follicle-enclosed oocytes, whereas depletion of GPR3 lowered intracellular cAMP levels and enhanced oocyte maturation. hCG treatment of isolated *Xenopus* ovarian follicles triggers metalloproteinase-mediated cleavage and inactivation of GPR3 (Deng et al., 2008, 2009). Also, it was found that a fraction of the classical PGR displays membrane localization (Martinez et al., 2007) and complexes with Gβγ subunits via the scaffold protein called the modulator of non-genomic steroid responses (NMAR), which may mediate steroid-triggered oocyte maturation (Haas et al., 2005). Furthermore, evidence has been presented that a G protein-coupled membrane progestin receptor XmPRβ expressed on the oocyte plasma membrane may be a physiological PGR involved in initiating the resumption of meiosis during maturation of *Xenopus* oocytes (Zhu et al., 2003; Josefsberg Ben-Yehoshua et al., 2007). Also, it was found that the classical androgen receptor (AR) is expressed in *Xenopus* oocytes. It can bind both androgens and P4 with high affinity. A minor fraction of the receptor is membrane-localized and suggested to mediate non-genomic effects of various steroid during ovulation (Lutz et al., 2001, 2003; Evaul et al., 2007).

In mammals, it was reported that the fastest effect of LHCGR activation observed *in vitro* is a decrease in cGMP in somatic follicular cells. This leads to cGMP diffusion out of oocytes and releases PDE3A inhibition, resulting in a decrease of intra-oocyte cAMP content and triggering oocyte maturation (Norris et al., 2009; Vaccari et al., 2009; Shuhaibar et al., 2015). Another early effect of LH that occurs within minutes in cultured mouse follicles is the closure of gap junctions. This is thought to be necessary for meiotic resumption because it restrains intercellular communications and ensures autonomous mode of oocyte maturation. It was demonstrated in mammals that EGFR and the MAPK pathway participate in gap junction closure (Prochazka and Blaha, 2015).

The synthesis of several proteins from maternal mRNA is required for MPF activation during progesterone-induced oocyte maturation. It was found that protein synthesis is triggered by cytoplasmic polyadenylation of maternal mRNAs mediated by the cytoplasmic polyadenylation element (CPE) present in their 3′ UTRs (Stebbins-Boaz et al., 1996; Mendez et al., 2000; Belloc and Méndez, 2008). A recent genome-wide analysis of the *Xenopus* oocyte transcriptome indicated that a family of U-rich sequence elements was enriched near the polyadenylation signal of transcriptionally activated mRNAs (Yang et al., 2020). The main newly synthesized proteins include cyclins, RINGO and Mos. Site-specific phosphorylation of CPEB catalyzed by Eg2, a member of the Aurora family protein kinases, was shown to be essential for the polyadenylation of c-mos mRNA (Andresson and Ruderman, 1998; Mendez et al., 2000). Cyclins and RINGO directly bind and activate Cdk1 kinase, whereas Mos induces MPF activation by activating the MAPK cascade (Figure 3). Notably, inhibition of protein synthesis prevents MPF activation induced by PKA inhibitors, that places protein synthesis downstream of PKA and upstream of MPF. The molecular link between PKA inactivation and *de novo* protein synthesis remains elusive. Although Mos synthesis starts soon after progesterone stimulation, the amount of Mos protein remains low until pre-GVBD, when it is stabilized by direct phosphorylation on Ser3 by activated MAPK (Matten et al., 1996). Several enzymes of protein phosphorylation, which represent two major signaling pathways, become prominently activated at that time, leading to a dramatic shift in the equilibrium between inactive pre-MPF and active MPF. A balance between the dual-specific Cdk1-activating protein phosphatase Cdc25C and Cdk1-inactivating protein kinases Myt1 and Wee1 controls this equilibrium through the phosphorylation state of the regulatory residues Thr14 and Tyr15 in Cdk1. Markedly, CSF activation precedes MPF activation during progesterone-induced oocyte maturation, suggesting that the activated MAPK pathway operates early in maturation. MAPK response to progesterone or Mos in living oocytes displays a feature of ultrasensitivity, corresponding to the extraordinarily high Hill coefficient of at least 35 (Ferrell and Machleder, 1998). The MAPK cascade, including Mos, MAPKK and MAPK, responds little to a weak stimulus and becomes robustly activated over a narrow range of stimulus concentration, resulting in a sigmoid stimulus-response curve, resembling that of a cooperative enzyme. This feature helps to maintain the prophase diplotene arrest in immature oocytes and ensures highly efficient all-or-none physiological response observed upon oocyte maturation (Ferrell, 1999).

Cytostatic factor activity first appears in progesterone-treated oocytes at the time of Mos accumulation. Activated MAPK phosphorylates and activates the downstream Ser/Thr-specific kinase Rsk, which downregulates Cdk1 inhibitory kinase Myt1, promoting MPF activation (Mueller et al., 1995b; Palmer et al., 1998). Mos was also shown to target Myt1 independently of MAPK (Peter et al., 2002). Another Cdk1-inactivating kinase, Wee1, is absent from immature oocytes and is synthesized later during maturation (Nakajo et al., 2000). In addition, activated MAPK promotes phosphorylation and activation of Plx1 kinase and Cdc25 phosphatase in the polo-like kinase pathway mediated by xPlkk1 and Plx1 protein kinases (Kumagai and Dunphy, 1996; Qian et al., 1998b). It was found that Plx1 is activated during oocyte maturation simultaneously with Cdc25 (Qian et al., 1998a). Once activated above a threshold level, MPF accumulates rapidly even in the absence of protein synthesis by an autocatalytic mechanism that involves several positive feedback loops (Figure 2). First, in the polo-like kinase Cdk1-activating pathway, Plx1 and Cdc25C can be directly phosphorylated and upregulated by Cdk1 (Abrieu et al., 1998). Second, active MPF was shown to increase Mos stability by direct phosphorylation on Ser3 (Castro et al., 2001). Third, MPF activates the MAPK cascade by upregulating Mos synthesis, probably, by increasing polyadenylation of maternal Mos RNA (Paris et al., 1991). Of note, activated MAPK is also capable of stimulating polyadenylation of Mos mRNA (Howard et al., 1999). It was found that the adaptor protein paxillin in cooperation with embryonic PolyAdenylation Binding Protein (ePABP) enhance, in an MAPK-dependent fashion, Mos translation and promote oocyte maturation (Miedlich et al., 2017). Thus, numerous interlocking positive feedback loops contribute to MPF amplification and stabilization in maturing *Xenopus* oocytes.

Soon after the complete MPF activation, dissolution of the oocyte nuclear membrane, or GVBD, occurs that can be easily detected by appearance of a white spot on the oocyte’s pigmented animal hemisphere. Breakdown of the nuclear envelope, a hallmark of M-phase entry, is triggered by Cdk1-mediated phosphorylation of nuclear lamins and other components of the nuclear envelope, resulting in their disassembly (Marshall and Wilson, 1997). Besides, MAPK is involved in M-phase chromosome condensation and cytoskeleton reorganization. Active MAPK localizes to spindle and centrosomes and induces interphase-metaphase transition of microtubule arrays (Gotoh et al., 1991; Verlhac et al., 1993). There is no real M-phase exit between the two meiotic divisions in *Xenopus* oocytes; interphase nuclei are not formed, and chromatin remains condensed. This prevents DNA replication between meiosis I and meiosis II and leads to generation of haploid gametes by the end of meiosis. The active MAPK cascade plays a key role in the meiotic suppression of DNA replication because ablation of endogenous Mos or pharmacological inhibition of MAPK activity in maturing oocytes allow premature DNA replication after meiosis I (Furuno et al., 1994; Gross et al., 2000). Although Cdk1 activity decreases after meiosis I due to a partial degradation of cyclin B, this decrease is incomplete and transient (Iwabuchi et al., 2000). The remaining activity of Cdk1 is required for normal meiotic transition, and inhibition of MPF assembly brings about premature DNA replication after the first meiotic M-phase (Picard et al., 1996; Iwabuchi et al., 2000). The activated MAPK cascade inhibits cyclin B degradation after MI and helps to maintain MPF activity between the two meiotic divisions by suppressing the ubiquitin-dependent anaphase-promoting complex (APC/C) via Rsk (Gross et al., 2000; Figure 3).

### Meiotic Metaphase Arrest

Maturing frog oocytes enter meiosis II with high activity of MPF and CSF and arrest again in the metaphase of the second meiotic division. These completely matured *Xenopus* oocytes, conventionally called “eggs” in frog and some other species, remain arrested at metaphase II by the high activity of MPF and CSF. Multiple intracellular signaling pathways assure stability of the meiotic metaphase arrest.

Cyclin degradation is greatly inhibited in the mature oocytes (Murray et al., 1989; Sagata et al., 1989). In the activated MAPK pathway, the MAPK downstream target, Rsk, directly phosphorylates and activates the inhibitors of the APC/C ubiquitin ligase, Emi2/Erp1 and Bub1, controlling cyclin B degradation (Schwab et al., 2001; Inoue et al., 2007; Nishiyama et al., 2007; Figure 3). Although the Emi2/Erp1 protein is absent from immature prophase-arrested oocytes, it accumulates in mature metaphase-arrested eggs, due to cytoplasmic polyadenylation and translational unmasking of its mRNA (Tung et al., 2007). The phosphorylated inhibitor proteins suppress ligase activity of the APC/C ubiquitin ligase by sequestering the Cdc20 activator subunit of APC/C (Shoji et al., 2006). The Cdk1-inactivating kinase Myt1 is kept inhibited by high activity of the MAPK pathway, and the Wee1 kinase is also suppressed through a phosphorylation-dependent mechanism in the metaphase-arrested eggs (Mueller et al., 1995a,b; Palmer et al., 1998). On the other hand, active MPF upregulates Mos synthesis via polyadenylation of mos mRNA (Howard et al., 1999) and increases stability of the Mos protein by direct phosphorylation on Ser3 (Castro et al., 2001).

Furthermore, both Cdk1 and MAPK activate the polo-like kinase pathway related to upregulation of the MPF-activating phosphatase Cdc25C (Kumagai and Dunphy, 1996; Qian et al., 1998b; Figure 3). In addition, the major anti-Cdk1 phosphatase PP2A-B55, which dephosphorylates Cdk1-phosphorylated substrates, is suppressed via the Greatwall kinase (Gwl) activated downstream of Cdk1 (Yu et al., 2006; Castilho et al., 2009; Vigneron et al., 2009; Figure 3). Activated Gwl phosphorylates the inhibitor proteins of PP2A-B55, such as ARPP-19 and/or α-endosulfine, which then directly interact with the phosphatase (Gharbi-Ayachi et al., 2010; Mochida et al., 2010). It was found that the Gwl/ARP19/PP2A-B55 pathway acts as a major component of MPF auto-amplification, that contributes to upregulation of MPF activity (Hara et al., 2012; Dupre et al., 2013). Notably, transcriptome-wide analysis demonstrated that massive deadenylation and degradation of mRNA occurs in *Xenopus* eggs at meiosis II (Meneau et al., 2020; Yang et al., 2020). It was found that a significant fraction of egg transcripts contained, in addition to CPEs, (A + U)-rich elements (ARE), characteristic of the mRNAs regulated by deadenylation, and suggested that opposing activities of CPEs and AREs define the fate of egg mRNAs (Belloc and Méndez, 2008).

Thus, after completion of ovulation, frog eggs are matured and arrested at metaphase II. Multiple mechanisms stabilize the meiotic metaphase arrest. The arrest allows eggs to await fertilization, preventing parthenogenesis after completion of meiosis. It was reported that unfertilized *Xenopus* eggs spontaneously exit the metaphase arrest and degrade by a well-defined apoptotic process within 48–72 h after completion of ovulation (Du Pasquier et al., 2011; Tokmakov et al., 2011).

## Follicular Rupture

Liberation of oocytes from ovarian follicles due to follicular rupture is one of the main and indispensable events of ovulation. It was found, using the frog model, that in contrast to hormone-mediated maturation, which is non-genomic, i.e., transcription-independent, *de novo* transcription seems to be necessary for follicular rupture (Bayaa et al., 2000; Lutz et al., 2003; Liu et al., 2005). It was demonstrated in different animals that follicular rupture is synchronized with maturation and it occurs within several hours after meiotic resumption (reviewed in Richards and Ascoli, 2018). The following section of our review focuses on the mechanisms of follicular rupture. The process has been investigated in different species, most thoroughly in mammals, with a help of various *in vitro* and *in vivo* growth and ovulation models and *in vitro* follicle cultures capable of reproducing main events of the ovarian cell cycle.

Three principal theories on the mechanism of follicular rupture were under consideration since long ago, including the smooth muscle theory, the intra-follicular pressure theory, and the proteolytic enzyme theory (Rondell, 1970; Espey, 1974). The involvement of protease activity in ovulation was initially suggested by Schochet (1916). Afterward, the proteolytic mechanism has been proved to play a predominant role in follicular rupture based on numerous experimental observations, including degradation of extracellular matrix at the follicular apex, LH or hCG-induced up-regulation of protease activity, and impeding oocyte release from ovarian follicles with protease inhibitors.

### Mammals

The role of proteolytic activity in follicular rupture was corroborated in mammals. It was clearly demonstrated that collagenous layers at the follicular apex become loosened and degrade before follicle rupture (Espey, 1967). Correspondingly, subsequent biochemical analyses revealed a preovulatory increase in ovarian collagenolysis *in vivo* and an increase of collagenase activity *in vitro* (Tsafriri et al., 1990; Reich et al., 1991; Murdoch and McCormick, 1992). It was found that multiple metalloproteinases and serine proteases are involved in decomposition of the follicle wall during ovulation. Specifically, members of the matrix metalloproteinase (MMP) family, including collagenases, gelatinases, stromelysins and membrane-type MMPs, tissue inhibitors of metalloproteinases (TIMPs), plasmin/plasminogen activator (PA) system, and the ADAM/ADAMTS family were implicated in follicle rupture during ovulation in mammals (reviewed by Duffy et al., 2019). Gonadotropins, such as LH and hCG, were demonstrated to induce both expression and activity of MMP and ADAMTS in the ovarian follicles. In monkeys and humans, upregulation of collagenases (MMP1), gelatinases (MMP2 and MMP9), stromelysins (MMP7 and MMP10), ADAMTSs (ADAMTS1, ADAMTS4, ADAMTS9, ADAMTS15), as well as the expression of TIMPs, such as TIMP1 and TIMP2, were shown to be induced by LH/hCG (Chaffin and Stouffer, 1999; Curry and Osteen, 2003; Peluffo et al., 2011). Several studies have proposed that the PA system also plays a key role in the degradation of the follicular wall during ovulation in mammals (Ny et al., 2002; Liu et al., 2004; Ohnishi et al., 2005). Plasminogen activators of the two types, urokinase type (PLAU) and tissue type (PLAT), are thought to be involved in the ovulatory process (Tsafriri et al., 1990; Blasi, 1993; Ebisch et al., 2008). In addition, the specific inhibitors of PA system, such as SERPINE_3_, SERPINB_2_, SERPINA_5_ and nexin I, are also induced in the ovary during ovulation (Aertgeerts et al., 1994; Ebisch et al., 2008). It was noted that proteolytic enzymes involved in follicular rupture are largely redundant; their substrate specificities are greatly overlapping, and no single proteolytic enzyme seems to be indispensable for this process (Duffy et al., 2019).

The primary targets of LH and gonadropin are the follicle cells expressing LHCGR, such as granulosa and theca cells, which surround an oocyte within a follicle. Multiple intracellular signal transduction pathways are triggered in mammalian follicle cells by activation of LHCGR (Figure 2, see section “Overview of Ovulation”). The signaling pathways involved in the activation of proteolytic enzymes during ovulation are not clearly understood. It was found that activation of the MAPK pathway in granulosa cells early in the ovulatory process is absolutely required for both oocyte maturation and follicle rupture, as demonstrated by the infertility phenotype of mice with ERK1 and ERK2 knockout in granulosa cells. The ERK1/2 knockout leads to a complete failure to ovulate and synthesize P4 (Fan et al., 2009). ERK activation in granulosa cells was shown to occur due to the release of tonic inhibition imposed by DUSP6 phosphotyrosine phosphatase on MEK1 (Law et al., 2017). It appears that ERK activation promotes steroidogenesis primarily via the StAR. Accordingly, ERK activation was shown to increase StAR expression in some steroidogenic cell lines (Gyles et al., 2001). Several downstream targets of ERK1/2 were found to be essential for follicular rupture. Especially, induction of the two transcription factors, CCAAT enhancer–binding protein (CEBP) α and β, that are highly important for ovulation, has been shown to depend on the MAPK pathway (Fan et al., 2009). Similarly to the ERK1/2 knockout, the combination of the granulosa-specific CEBPaα and CEBPβ knockouts leads to a complete failure to ovulate (Fan et al., 2011).

Another important signaling molecule that is engaged downstream of the LH receptor is the EGF receptor (EGFR). Several studies demonstrated that EGFR signaling can promote steroidogenesis in gonadal cells, including Leydig cells and ovarian follicles, by upregulating StAR (Manna et al., 2002; Jamnongjit and Hammes, 2006). Activation of EGFR seems to be necessary for gonadotropin-induced steroid production in preovulatory follicles, as inhibition of the EGFR protein kinase activity completely abolishes LH-triggered production of P4 (Jamnongjit et al., 2005). In addition, inhibition of EGFR kinase activity significantly suppresses hCG-induced meiotic maturation and follicle rupture, so that oocytes remain arrested at the diplotene stage of the first meiotic prophase in intact ovarian follicles (Ashkenazi et al., 2005). It appears that EGFR activation during ovulation is mediated by the MAPK pathway. In mammalian follicles, activated ERK1/2 mediate transcriptional induction of the EGF–like ligands, such as amphiregulin, epiregulin, and betacellulin, in granulosa cells (Park et al., 2004). These factors function as autocrine and paracrine activators of EGFR on theca and granulosa cells, promoting increased StAR activity, presumably via phosphorylation, and subsequent steroid production. Remarkably, incubation of follicles with the EGF-like ligands evokes the morphological and biochemical events of ovulation, such as oocyte maturation and cumulus expansion (Park et al., 2004).

Progesterone receptor, a nuclear receptor transcription factor, also plays an important role in follicular rupture. It is robustly induced in mural granulosa cells via protein kinase A/cAMP response element-binding protein (CREB)-mediated transactivation and MAPK pathway activation after the LH surge (Park and Mayo, 1991). The key role of PGR in follicular rupture was confirmed in rodents, monkeys, and humans, where inhibition of P4 synthesis or its activity prevented ovulation (Espey et al., 1990; Lydon et al., 1995; Hibbert et al., 1996; Bishop et al., 2016). The precise mechanism by which PGR promotes follicle rupture is unknown, however there is mounting evidence for a link between P4 and upregulation of MMPs and other proteases (Robker et al., 2000; Chaffin and Stouffer, 2002). During ovulation, PGR is acutely activated in the presence of high local concentrations of P4 and translocated into the nucleus, where it initiates transcription of the proteins, such as ADAMTS-1 and cathepsin L, essential for follicular rupture. Knockout of PGR gene results in a complete and specific block of ovulation, however, cumulus expansion and oocyte meiotic maturation occur normally, and oocytes extracted from the knockout follicles can be fertilized (Robker et al., 2000), suggesting a specific role of PGR in mediating follicle rupture. Accordingly, pharmacological inhibition of PGR was reported to block ovulation (Jesam et al., 2016; Schutt et al., 2018). These findings are consistent with the idea that P4 is required for follicular rupture in mammalian species.

Several findings suggested involvement of prostaglandins (PGs) upstream of follicular rupture. It was demonstrated that inhibitors of eicosanoid synthesis could block ovulation and the LH/hCG-induced rise in ovarian collagenolysis (Reich et al., 1991). Synthesis of prostaglandin precursors during ovulation is governed by cyclooxygenase 2 (COX2), also known as prostaglandin synthase 2 (PTGS2). The enzyme is strongly induced in rat, primate and human preovulatory follicles after the LH surge (Sirois et al., 1992, 2004; Stouffer et al., 2007). It was shown that, COX2 knockout mice, which produce no PGs, have reduced rates of ovulation, defected cumulus expansion, and infertility. Ovulation can be restored in the COX2-deficient mice by prostaglandin E2 (PGE2) (Lim et al., 1997; Davis et al., 1999). The inhibitors of COX2 activity, such as meloxicam, also prevent ovulation, suggesting their use as emergency contraceptives (McCann et al., 2013). Although the predominant prostaglandin produced by granulosa cells is PGE2, some other PGs, such as PGF2α, have been shown to mediate certain aspects of follicular rupture (Robker et al., 2018).

Recent studies demonstrated that vasoconstriction at the follicle apex plays an essential role in mammalian follicle rupture. Vasoconstriction of apical vessels was shown to occur within several hours preceding follicle rupture in wild-type mice, and the ovulatory follicles from the mice with defective vasoconstriction failed to rupture (Migone et al., 2016). It was found that endothelin-2 (Edn2), a short vasoconstrictor peptide that is transiently produced by granulose cells before follicle rupture (Palanisamy et al., 2006), is indispensable for ovarian contraction. Edn2 knockout resulted in a significant reduction in the number of ovulated oocytes (Cacioppo et al., 2017). Also, pharmacological inhibition of the preovulatory Edn2 induction, that can be reversed with the exogenous peptide, or blocking Edn2 receptors were shown to prevent vasoconstriction and follicle rupture (Migone et al., 2016).

Of note, in contrast to frogs, mammalian oocytes are ovulated in a complex with cumulus cells, a specific sublineage of granulosa cells directly wrapping the oocyte. Expansion of cumulus cells that occurs after initiation of meiosis but before follicular rupture represents an important step of mammalian ovulation regulated via a distinct intracellular pathway (Figure 2). It involves MAPK-mediated transcriptional induction of the EGF-like ligands, such as amphiregulin, epiregulin, and betacellulin in granulosa cells (Park et al., 2004). The EGF-Ls secreted by granulosa cells transactivate EGFR on cumulus cells, inducing the production of cumulus matrix proteins, such as hyaluronan synthase 2 (HAS2) and pentraxin 3 (PTX3), which cause cumulus oocyte complex (COC) expansion. It was found that inhibition of cumulus cells expansion also impedes follicular rupture, suggesting that the two processes engage overlapping signaling pathways with similar changes in gene expression. For instance, PGs were reported to affect mammalian ovulation through their influence on cumulus expansion (Sirois et al., 2004). In detail, molecular mechanisms of coordination through the cumulus complex are reviewed elsewhere (Russel and Robker, 2007; Robker et al., 2018).

### Drosophila

Several parallels were revealed between *Drosophila melanogaster* and mammalian ovulation, including follicle rupture, at both cellular and molecular levels. An *ex vivo* follicle rupture assay has been developed that allowed direct quantification of follicles’ capacity to respond to ovulation stimuli and rupture in *Drosophila* (Deady and Sun, 2015). It was found, using both *in vivo* and *in vitro* approaches, that posterior follicle cells surrounding mature oocytes are selectively degraded during ovulation in *Drosophila*. Like in mammals, follicle rupture also depends on MMP2 activity localized at the posterior end of mature follicles (Deady et al., 2015). It was shown that MMP2 activity is regulated by the octopaminergic signaling in mature follicle cells. Exogenous octopamine is sufficient to induce follicle rupture when isolated mature follicles are cultured *ex vivo*, in the absence of the oviduct or ovarian muscle sheath. Accordingly, knocking down the octoampine receptor Oamb in mature follicle cells prevented OA-induced follicle rupture *ex vivo* and ovulation *in vivo*. It was further found that follicular octopamine signaling induces MMP2 enzymatic activation but not MMP2 protein expression, likely via intracellular Ca2+ (Deady and Sun, 2015). The developed *ex vivo* follicle rupture assay was proposed to query enzymatic activity and to perform genetic or pharmacological screens to identify genes or small molecules involved in follicle rupture in *Drosophila* (Knapp et al., 2018). It was further demonstrated that steroid signaling in mature follicle cells is important for *Drosophila* ovulation. Knocking down the shade monooxygenase that converts ecdysone to its active analog 20-hydroxyecdysone or disruption of the ecdysone receptor specifically in mature follicle cells, was shown to block follicle rupture (Knapp and Sun, 2017). Furthermore, it was demonstrated that ecdysteroid signaling is essential for proper activation of the MMP MMP2 and follicle rupture. The zinc-finger transcription factor Hindsight (Hnt) has been implicated in the development of ovulation competency of Drosophila follicles. This factor is upregulated in mature follicle cells. It was found that Hnt elevates MMP2 expression in posterior follicle cells and expression of the adrenergic receptor Oamb in all follicle cells, which is essential for follicle rupture and ovulation (Deady et al., 2017).

### Fish Models

Follicle rupture during ovulation has been thoroughly investigated in fish models, most notably, in teleosts (bony fishes) (reviewed by Takahashi et al., 2019). In teleost fishes, ovulation results in discharge of fertilizable oocytes from ovarian follicles into the ovarian or abdominal cavity. Several studies focused on the involvement of proteases and protease inhibitors in this process, and it was found that robust proteolytic events accompany ovulation in various teleost fishes. For instance, it was shown that collagenolytic activity is present and it increases prior to ovulation in follicle walls of brook trout (*Salvelinus fontinalis*) and yellow perch (*Perca flavescens*) follicles, suggesting involvement of the MMP activity in digesting the follicle wall (Berndtson and Goetz, 1988, 1990). Involvement of kallikrein-like serine protease KT-14 in trout ovulation was also demonstrated (Hajnik et al., 1998). It was further demonstrated that LH-induced *in vitro* ovulation of brook trout follicles involves follicle contraction and activation of multiple proteolytic genes (Crespo et al., 2013). LH increased mRNA expression levels of MMPs, such as MMP2 and MMP19, and other enzymes with proteolytic action during ovulation, such as disintegrin, ADAMTS1 and plasminogen, in brook trout preovulatory follicles and during the progression of LH-induced ovulation. Of note, the expression of tissue inhibitor of the MMP TIMP2 paralleled that of MMP2, suggesting the existence of a controlled mechanism of MMP action (Crespo et al., 2013). More recently, genome-wide studies identified the genes upregulated and downregulated in the ovulatory follicle during ovulation in fish (Bobe et al., 2006; Klangnurak and Tokumoto, 2017; Liu et al., 2017). Serine protease 23 and metalloprotease ADAM22 were found to be induced in the periovulatory follicle of rainbow trout (Bobe et al., 2006). A significant change in the expression of ADAMTS8b, ADAMTS9 and MMP9 was observed in preovulatory follicles of zebra fish (Liu et al., 2017). It was accompanied by upregulation of the intrinsic proteinase inhibitors, such as Serpine 1 and TIMP2.

The detailed studies on follicle rupture during ovulation in fishes were carried out using the freshwater teleost medaka (*Oryzias latipes*). *In vitro* ovulation models developed for medaka allowed identification of the hydrolytic enzymes responsible for oocyte liberation during ovulation. Furthermore, similarly to mammals, the involvement of prostaglandin E2, PGE2, cyclooxygenase-2, and PGE2 receptor subtype EP4b in medaka ovulation has also been demonstrated (Takahashi et al., 2013). It was found that follicle rupture in the medaka ovary involves the cooperation of at least three different MMPs, and the tissue inhibitor of MMP-2b protein (Ogiwara et al., 2005). The discrete roles of each of these proteins in follicle rupture has been elucidated. It was found indicated that gelatinase A (MMP2) induces the hydrolysis of type IV collagen constituting the basement membrane, membrane-type 2 MMP (MMP15) degrades type I collagen present in the theca cell layer, whereas MT1-MMP (MMP14) and the tissue inhibitor of MMP-2b TIMP2b are involved in production and regulation of gelatinase A (Ogiwara et al., 2005). Consistently, it was found, using an *in vitro* ovulation model based on the whole ovary culture, that medaka ovulation could be inhibited by the addition of metalloproteinase inhibitors to the ovarian culture (Ogiwara et al., 2010). In addition, involvement of a plasmin-like protease in early stage follicle rupture during ovulation in medaka has been demonstrated (Ogiwara et al., 2012). The enzyme was shown to participate in the rupture several hours before the activation of MMP-mediated hydrolysis. Furthermore, involvement of urokinase plasminogen activator and plasminogen activator inhibitor-1 in follicle rupture during ovulation in medaka has been shown (Ogiwara et al., 2015). It was found, using an *in vitro* ovulation assay, that various serine protease inhibitors, including a specific plasmin inhibitor, significantly reduce ovulation rates. A follicle rupture model involving two different proteolytic enzyme systems, serine protease and MMP, and sequential two-step hydrolysis of extracellular matrix has been proposed in medaka ovulation (Ogiwara et al., 2012).

Presently, molecular mechanisms leading to upregulation of the multiple proteolytic enzymes in teleosts during ovulation have not been well investigated. It was reported that ovarian prostaglandin synthesis greatly increases in several teleost species during ovulation, and that a non-selective cyclooxygenase inhibitor indomethacin blocks both *in vivo* and *in vitro* ovulation, suggesting that prostaglandins play an important role during ovulation in these oviparous species (Takahashi et al., 2013). Most recently, the involvement of Cdk activity in degradation of the extracellular matrix during ovulation in medaka has been demonstrated (Ogiwara and Takahashi, 2019). It was found that the Cdk inhibitor roscovitine inhibited both follicle ovulation and follicular expression of MMP15, implicating Cdk activity in the expression of this protease. It was further suggested that the nuclear progestin receptor, PGR, which is induced by the LH surge, serves as a functional transcription factor for MMP15 expression after Cdk9/Cyclin I-mediated phosphorylation (Ogiwara and Takahashi, 2019). Notably, in contrast to mammalian species, the importance of MAPK activation for follicle rupture in teleosts has not been established. Moreover, it was reported that incubating medaka follicles with a MEK inhibitor did not lead to significant changes in PGR phosphorylation (Ogiwara and Takahashi, 2019).

### Frogs

Paradoxically, although oocyte maturation was most thoroughly investigated in amphibians, little is known about the mechanisms of follicle rupture in frog species, including *Xenopus laevis.* Most notably, the involvement of proteolytic enzymes in follicle rupture has not been established and intracellular signal transduction pathways leading to their activation were not identified. In fact, relatively few studies have addressed mechanisms of follicle rupture during ovulation in frogs. This situation can be attributed, in part, to the lack of reliable *in vitro* reconstitution of follicle rupture in the frog model and to difficulties of dissecting this process in living animals.

In frogs, *in vitro* maturation and ovulation of *Rana pipiens* and *Rana dybowskii* oocytes was observed in the ovarian fragments and isolated follicles treated with homologous pituitary extracts (Wright, 1961; Dettlaff et al., 1964; Smith et al., 1968). It was reported that oocyte liberation can be achieved more readily from ovarian fragments than from isolated ovarian follicles and hypothesized that attachment of follicles to a site in the ovarian fragment is beneficial for contraction of follicle wall during oocyte extrusion (Schuetz, 1986; Kwon et al., 1992). The efficiency of pituitary preparations varied considerably, exhibiting seasonal dependence; ovarian fragments from some animals failed to ovulate in response to any treatment and spontaneous follicular rupture occurred in cultured ovarian fragments collected during some seasons of the year (Smith et al., 1968; Kwon et al., 1992). It was also reported that maturation proceeded in the absence of follicle rupture in follicles isolated from some animals. Moreover, follicle rupture in the *in vitro* cultured ovarian fragments lagged greatly behind GVBD, whereas these two events occurred almost simultaneously during natural ovulation. Furthermore, although various steroid hormones were found to be effective in inducing *in vitro* maturation of isolated frog oocytes, the hormones failed to promote follicular rupture (Subtelny et al., 1968; Schuetz and Lessman, 1982). Early studies of *in vitro* frog ovulation demonstrated that intact follicles of *Rana pipiens* ovulated in response to pituitary homogenate (FPH) due to, in part, mechanical contractions of the follicle wall (Schuetz and Lessman, 1982). Ovulation and follicular contractions could not be observed following removal of the surface epithelium in the presence of the intact thecal layer. Treatment of isolated amphibian ovarian follicles with FPH was shown to increase follicular P4 levels and initiate oocyte maturation (Petrino and Schuetz, 1987). However, it was found that oocytes, regardless of the removal of any or all follicular wall layers, matured but did not ovulate in response to P4. These results provided functional evidence for a role of the ovarian surface epithelium and demonstrated that P4 alone is not sufficient to initiate follicle rupture during ovulation in frogs. Nevertheless, in the later studies using cultured ovarian fragments to investigate release of frog oocytes from follicles during ovulation, follicle rupture has been observed in the vigorously shaken ovarian fragments after extensive overnight incubation in the presence of P4 or hCG (Liu et al., 2005; Sena and Liu, 2008).

At present, signaling pathways involved in the process of follicle rupture in frogs have not been established. Importance of MAPK activation in follicle cells, which is a prerequisite for follicle rupture in mammals, was not explicitly demonstrated, and the transcription factors involved in the upregulation of follicular protease activity during ovulation were not identified. Involvement of PKC was suggested by the findings that TPA effectively stimulated PGF_2_α secretion and ovulation, and that PKC inhibition dramatically suppressed FPH- or TPA-induced PGF_2_α secretion and ovulation observed in the isolated ovarian fragments of *Rana pipiens*. It was further demonstrated that PKC mediates gonadotropin-induced production of PGF_2_α but not steroid synthesis in the frog ovaries (Chang et al., 1995). It was reported that exposure of *in vitro* cultured ovarian follicles to PGF_2_α stimulated low levels of follicle rupture in the absence of maturation (Schuetz, 1986; Chang et al., 1995). Addition of PGF_2_α, but not PGE, to cultured follicles markedly enhanced the incidence of ovulation in follicles exposed to P4 or pituitary homogenates. Ovulatory effects of PGF_2_α were suggested to be mediated through the follicular epithelium. Similarly, *in vitro* studies of oocyte ovulation using ovarian fragments isolated from the toad *Bufo arenarum* revealed that PGF2α, but not PGE1, increased ovulatory responses to either pituitary homogenate or P4 at suboptimal doses, indicating that PGF2α exerted a synergistic potentiating effect (Ramos et al., 2008). Furthermore, it was found that the transcriptional inhibitor actinomycin D blocked P4-induced follicle rupture, however, PGF_2_α was found to overcome the inhibitory effect of the drug, indicating that this prostaglandin has the ability to induce follicle rupture in the absence of protein synthesis (Liu et al., 2005; Sena and Liu, 2008). Notably, the follicular levels of both PGF_2_α and PGE_2_ increase during the preovulatory period, however PGF_2_α levels are much higher than that of PGE_2_. It was found that expression of COX2 but not COX1 is up-regulated during hCG- or P4-induced ovulation *in vitro*, leading to increased synthesis of PGF_2_α during preovulatory period (Sena and Liu, 2008). It was found that a PGR antagonist RU486 inhibits COX2 expression and PGF_2_α synthesis, suppressing, in a dose-dependent manner, P4-induced ovulation observed in the isolated *Xenopus* ovarian follicles (Dhillon et al., 2010). It was suggested that although PGF_2_α is necessary for P4-induced ovulation of *Xenopus* oocytes, it may not be essential for hCG-induced ovulation (Sena and Liu, 2008). Based on the above findings, it has been hypothesized that ovulation and maturation of amphibian oocytes can be mediated by separate classes of hormones, and that normal synchronization of ovulation and maturation requires the combined action of prostaglandins and steroids within different follicular compartments.

**FIGURE 4:**
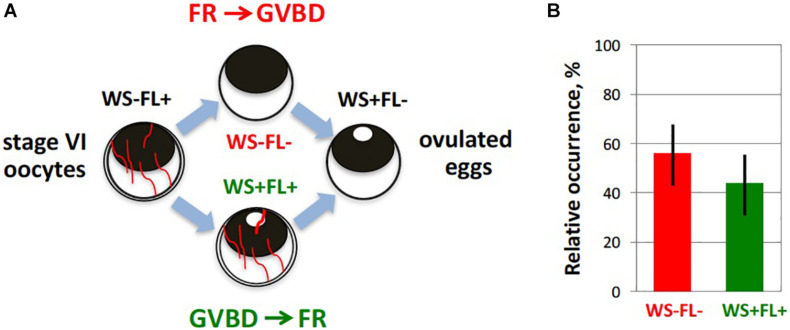
Timing of maturation and follicle rupture during *Xenopus* oocyte ovulation. Progression of meiotic maturation in the developed *in vitro* ovulation model (Tokmakov et al., 2019) was judged by appearance of a white spot (WS) on the animal hemisphere of oocytes, reflecting germinal vesicle breakdown (GVBD), and occurrence of follicular rapture (FR) was defined by the loss of the follicle layer (FL). **(A)** Theoretically, two alternative scenarios, GVBD-FR and FR-GVBD, can occur during ovulation, as indicated in the figure. To distinguish between them, the four morphologically different types of cells were counted during hormone-induced ovulation. They included oocytes without WS surrounded by FL (WS-FL+), oocytes with WS and FL (WS + FL+), oocytes without WS and FL (WS–FL–), and oocytes with WS and without FL (WS + FL). The WS-FL + phenotype characterizes original oocyte population, and the WS + FL– phenotype corresponds to ovulated mature eggs. **(B)** Experimentally observed relative frequencies of the two intermediate phenotypes, WS + FL + and WS–FL–, are very close, indicating that GVBD and FR occur almost simultaneously.

Our recent study using a new *in vitro* ovulation model presented the first direct evidence for involvement of MAPK and MMP activities in follicle rupture during *Xenopus* oocyte ovulation (Tokmakov et al., 2019). It was found that both meiotic maturation and follicle rupture can be reproduced *in vitro* using isolated ovarian follicles shortly pretreated with collagenase before P4 administration. Oocyte maturation and follicle rupture could also be observed in the hCG-treated follicles, albeit with somewhat lower rates. Notably, follicular rupture occurred at about the same time as GVBD (Figure 4), and it didn’t occur in the absence of P4, indicating physiological relevance of the developed model. Indeed, the two processes are highly coordinated *in vivo*: mature oocytes are not retained in the ovary, and immature oocytes don’t ovulate during natural ovulation in frogs. Importantly, oocyte release from ovarian follicles, but not oocyte maturation, was inhibited in this model in the presence of a wide-specificity MMP inhibitor GM6001. In addition, pharmacological inhibition of the MAPK pathway using a specific MEK inhibitor U0126 was demonstrated to suppress both meiotic maturation and follicular rupture (Tokmakov et al., 2019). These findings prove that MAPK and MMP activities are involved in the process of oocyte liberation from ovarian follicles in frogs. Further studies are necessary to identify proteolytic enzymes engaged in follicle rupture and to delineate the pathway of MAPK activation in follicle cells during ovulation in frogs.

## On the Coordination of Maturation and Follicular Rupture

Oocyte liberation from the ovarian follicles and oocyte maturation are interdependent, tightly linked, and coordinated processes. All naturally ovulated frog eggs are matured and arrested in metaphase II with high activity of MPF and CSF, and all non-ovulated oocytes retained in the frog ovaries after hormonal stimulation remain immature and arrested in prophase I with low activity of MPF and CSF. Communication between the oocyte and follicular cells is necessary to coordinate the two major ovulation processes, meiotic maturation and follicle rupture, during normal physiological ovulation. However, overall coordination of ovulatory process in ovarian follicles of frogs is poorly studied. The complex intercellular communication follicular networks have been most thoroughly investigated in mammalian models and comprehensively reviewed in several recent publications (Richards and Ascoli, 2018; Robker et al., 2018; Duffy et al., 2019). Here, we provide just a bird’s-eye view of intrafollicular coordination during ovulation.

In the resting ovarian follicle, the oocyte and cumulus cells are directly connected by terminal gap junctions. The terminal gap junctions allow transit of small (<1 kDa) molecules between the oocyte and cumulus cell cytoplasm. Energy metabolites, such as glucose, pyruvate, and ATP can be transported from granulosa cells via gap junctions (Edry et al., 2006; Johnson et al., 2007). Gap junctions between oocyte and follicle cells have also been observed in frog follicles (Konduktorova and Luchinskaia, 2013), however, their role in the ovulatory process was not thoroughly investigated in this model. Importantly, cAMP and cGMP, which are needed to maintain diplotene arrest in immature oocytes (see section “Diplotene Arrest in Immature Oocytes” for details), are transported into oocytes from cumulus cells through the terminal gap junctions (Edry et al., 2006; Shuhaibar et al., 2015). The importance of direct communication between the oocyte and somatic cells in the follicle was revealed by knockout of connexin 37, a gap junction protein involved in the direct transfer of small molecules. It was found that connexin 37-deficient mice can produce large preovulatory follicles but fail to ovulate, indicating that normal ovulatory response is not possible without sustained communication between oocytes and surrounding somatic cells (Simon et al., 1997). It was demonstrated that activation of Gαs-coupled LH receptors stimulates adenylyl cyclase, elevates intracellular cAMP levels, activates PKA in follicular cells, and promotes steroidogenesis by increasing expression of the StAR in both theca and granulosa cells of mammalian follicle, as well as by StAR activation via its phosphorylation on serine 195 (Wood and Strauss, 2002; Jamnongjit and Hammes, 2006). Importantly, the LH surge causes closure of gap junctions, presumably through phosphorylation of the connexin proteins (Granot and Dekel, 1994). Involvement of the MAPK pathway and EGFR in the gap junction closure has been demonstrated in mammals (Norris et al., 2008, 2010; Prochazka and Blaha, 2015). MAPK activation and MAPK-mediated EGFR signaling in granulosa cells early in ovulation are indispensable for both oocyte maturation and follicle rupture, as detailed in the sections “Meiotic Resumption” and “Mammals” of this article. The MAPK pathway (Ras/Raf/MEK/ERK pathway) is triggered in granulosa cells by Src kinase via Gβγ proteins activated by G-protein coupled receptors (Jamnongjit and Hammes, 2006).

Discontinuation of junctional communications within the ovarian follicle promotes oocyte maturation (Sela-Abramovich et al., 2006). It is assumed that maturation proceeds in an entirely autonomous fashion after gap junction closure. The closure of gap junctions terminates transport of cAMP and cGMP from cumulus cells into the oocyte, leading to a decline in intraoocyte cAMP and cGMP levels. The decreased level of cGMP releases inhibition of phosphodiesterase 3A, further promoting accelerated degradation of cAMP and triggering meiotic resumption of the oocyte. In addition, high follicular levels of progestogens and/or androgens, which are synthesized by granulosa cells in response to LH, lead to further decrease in the intraoocyte level of cAMP due to G protein-coupled receptor-mediated inhibition of plasma membrane AC (see section “Meiotic Resumption” for details). Although the latter mechanism seems to be a major cause triggering meiotic resumption in frogs, P4 can also influence oocyte maturation in some mammalian species. For example, treatment with P4 agonists in the absence of gonadotropin was shown to promote oocyte maturation to metaphase II, but not ovulation, in rhesus macaque follicles (Borman et al., 2004). It should be emphasized that the steroid stimulates transcription-independent oocyte maturation via the membrane P4 receptor and transcription-dependent follicle rupture via the classical nuclear receptor.

Evidence is presented that P4 mediates follicular rupture in mammals. Inhibition of P4 synthesis or its activity prevents ovulation (Espey et al., 1990; Hibbert et al., 1996) and mice lacking nuclear P4 receptor do not ovulate (Lydon et al., 1995). Interestingly, the role of PGR in mammals seems to be confined mainly to mediating follicular rupture, because activation of cumulus expansion and oocyte meiosis occur normally, and the oocytes obtained from the P4 receptor-knockout follicles are fertilizable (Robker et al., 2000). Antagonists of PGR, such as mifepristone (RU486), valaprisan, ulipristal, and some others can effectively block ovulation and are used as contraceptives in clinics (Jesam et al., 2016; Schutt et al., 2018). The observation that P4 alone is able to promote the entire ovulation process in the isolated ovarian follicles of *Xenopus laevis* (Tokmakov et al., 2019) suggests that PGR may also be engaged in follicular rupture in frogs. Of interest, it was reported that in amphibians, unlike in mammals, RU486 acts as an agonist of P4 (Skoblina, 2002).

It appears that oocyte maturation and follicle rupture unfold completely independently after steroid production and gap junction closure following the gonadotropin surge. Indeed, the well-established fact that meiotic maturation can readily be induced by P4 and some other steroids in defolliculated frog oocytes, as well as the recent finding that blocking follicle rupture by means of MMP inhibition does not affect *Xenopus* oocyte maturation (Tokmakov et al., 2019), highlight autonomous character of oocyte maturation. However, it cannot be ruled out that oocytes affect *vice versa* ovulatory processes in follicle cells. Notably, oocytes and follicle cells are known to communicate via paracrine signals in addition to gap junctions (Eppig, 2001). For example, it was reported that expansion of the mouse cumulus oophorus requires a factor secreted by the oocyte, so called, the cumulus expansion enabling factor, CEEF (Buccione et al., 1990). Also, it was found that co-incubation of defolliculated mouse oocytes with isolated cumulus cells markedly increases MAPK activity in the cumulus cells stimulated by FSH, while MAPK activity remains low in the absence of oocytes, indicating that oocytes secrete some paracrine factors promoting MAPK activation in cumulus cells (Su et al., 2003). Oocyte-derived paracrine factors GDF9 and BMP15 were found to function in a cooperative manner to stimulate signaling pathways in preovulatory cumulus granulosa cells and to maintain the integrity of the cumulus-oocyte complex (Matzuk et al., 2002). Further evidence for the existence of an oocyte-granulosa cell regulatory loop has been provided by the observation that the BMP15 and GDF9 growth factors regulate MAPK activation in the cumulus cells and cumulus expansion, as well as meiotic oocyte resumption after the preovulatory LH surge (Su et al., 2004). A hypothesis of bidirectional communication between the oocyte and follicle cells has been proposed to corroborate coordination of meiotic maturation and cumulus expansion in the isolated cumulus cell-enclosed mouse oocytes (Su et al., 2003).

The existence of bidirectional communication between oocytes and follicle cells can easily explain the fact that follicular rupture is well synchronized with maturation and meiotic resumption, so that both processes naturally occur *in vivo* within several hours of hormonal stimulation. Moreover, it was demonstrated, using an *in vitro* model of *Xenopus* oocyte ovulation, that follicle rupture develops simultaneously with GVBD (Tokmakov et al., 2019; Figure 4), which takes place during the first meiotic metaphase. Paracrine signals from maturing oocytes to follicle cells would potentially prevent the failed reproductive scenario, where follicle rupture is initiated in the absence of meiotic maturation, leading to ovulation of immature and unfertilizable oocytes. On the other hand, this kind of feedback would help to avoid the situation where matured oocytes remain entrapped inside ovarian follicles in the ovary. It has been reported previously that unfertilized matured *Xenopus* oocytes die by a well-defined apoptotic process within 48–72 h of hormonal stimulation (Du Pasquier et al., 2011; Tokmakov et al., 2011; Iguchi et al., 2013). Eggs from different species, including mammals, have been shown to die by apoptosis in the absence of fertilization (Tokmakov et al., 2017, 2018). Clearance of large follicle-entrapped apoptotic eggs from the ovaries may represent a formidable homeostatic challenge that would potentially overload the immune system. In frogs, no matured follicular oocytes can be observed in the ovary after ovulation, and all oocytes found in the genital tract during ovulation have completed GVBD, suggesting that GVBD and oocyte liberation from ovarian follicles are remarkably synchronized and coordinated *in vivo*. However, a great number of eggs dying by an apoptotic process are retained in the frog genital tract after ovulation (Iguchi et al., 2013; Tokmakov et al., 2018; Sato and Tokmakov, 2020), indicating that oviposition rather than ovulation may be a bottleneck of the reproductive process in oviparous species.

## Conclusion

Using follicular fragments, isolated follicles, and denuded oocytes greatly facilitates dissection of the ovulatory process in different animals. A remarkable progress has been achieved in depicting intricate molecular mechanisms of meiotic maturation using frog oocytes and eggs. The studies of follicular rupture and the entire process of ovulation have been successful in several other species. The crucial follicular processes indispensable for successful ovulation, such as steroidogenesis, prostaglandin synthesis, and proteolysis, have been most thoroughly investigated in mammals and teleost fishes. However, coordination of maturation and follicle rupture, the two interdependent and highly synchronized ovulatory processes, as well as other follicular events, remains largely unexplained. Multiple endocrine, paracrine, and autocrine signaling pathways regulate ovulation, and their coordinated cooperation requires further investigation.

## Author Contributions

AT, VS, and K-IS conceived the review topic and discussed its content. AT wrote the manuscript. AT, VS, and K-IS revised the work. All the authors approved the final submitted version.

## Conflict of Interest

The authors declare that the research was conducted in the absence of any commercial or financial relationships that could be construed as a potential conflict of interest.
